# Molecular mechanisms and potential implications of ferroptosis, cuproptosis, and disulfidptosis in septic lung injury

**DOI:** 10.3389/fmed.2025.1615264

**Published:** 2025-08-15

**Authors:** Jiaxin Li, Han Liu, Zhitao Shan, Kezhuo Zhong, Qun Liang

**Affiliations:** ^1^The First Clinical Medical School, Heilongjiang University of Chinese Medicine, Harbin, China; ^2^University College London, London, United Kingdom; ^3^The First Clinical College, Heilongjiang University of Chinese Medicine, Harbin, China; ^4^Department of Intensive Care Medicine, First Affiliated Hospital Heilongjiang University of Chinese Medicine, Harbin, China

**Keywords:** sepsis, lung injury, ferroptosis, cuproptosis, disulfidptosis, iron metabolism, copper homeostasis, sulfide metabolism

## Abstract

Sepsis remains a life-threatening condition worldwide, causing significant morbidity and mortality across diverse patient populations. Among the various organs adversely affected by sepsis, the lung is particularly vulnerable, often succumbing to acute lung injury (ALI) or its more severe form, acute respiratory distress syndrome (ARDS). Recent basic and translational research has highlighted the importance of multiple regulated cell death (RCD) pathways beyond traditional apoptosis in the pathogenesis of septic lung injury. Three such RCDs, termed ferroptosis, cuproptosis, and disulfidptosis, are increasingly studied for their relevance to critical illnesses. Ferroptosis involves iron-driven lipid peroxidation, cuproptosis depends on copper ion imbalance and mitochondrial protein aggregation, and disulfidptosis emerges from dysregulated sulfide metabolism leading to excessive disulfide bond formation. This review provides an extensive discussion of these RCD pathways within the context of sepsis-induced lung injury. We begin by summarizing the current state of knowledge in septic lung injury, emphasizing inflammatory, immunological, and oxidative stress mechanisms. We then provide a detailed overview of ferroptosis, cuproptosis, and disulfidptosis, illustrating their molecular underpinnings and how they intersect with established sepsis pathways, such as tumor necrosis factor (TNF), nuclear factor kappa B (NF-κB), and mitogen-activated protein kinase (MAPK) signaling cascades. We also discuss emerging findings on the crosstalk among these RCD modes, potential biomarkers for early detection, and therapeutic targets for modulating these pathways. Although many of these findings remain in the early stages of translational research, they collectively underscore the complexity of septic lung injury and offer new directions for improving clinical management. Future investigations, bolstered by integrative “omics” approaches, refined animal models, and well-designed clinical trials, will be pivotal to fully realize the diagnostic and therapeutic potential of ferroptosis, cuproptosis, and disulfidptosis in sepsis. We further propose a “redox stress-metal homeostasis-sulfur metabolism” triangular network, centered on Nrf2’s dual regulation of iron/copper transporters and glutathione synthesis, as a unifying framework for RCD modulation in sepsis. A signaling interaction diagram highlights actionable targets for combinatorial therapies.

## Introduction

Sepsis is a severe, life-threatening syndrome arising from the host’s dysregulated immune response to infection, leading to tissue damage, organ failure, and high mortality rates (1–3). In recent decades, significant progress in recognizing sepsis as a heterogeneous condition has spurred the development of refined definitions and clinical guidelines. Even so, global mortality remains alarmingly high, ranging from 20% to over 40% depending on resource availability and comorbidities (4, 5). The lung is among the first and most frequently affected organs in sepsis, with sepsis-induced acute lung injury often precipitating respiratory failure, which can progress to acute respiratory distress syndrome (ARDS).

While apoptosis, necroptosis, and pyroptosis are well-documented in sepsis-induced organ injury, this review focuses on ferroptosis, cuproptosis, and disulfidptosis due to their emerging roles in septic lung damage and their unique mechanistic underpinnings. These pathways driven by iron/copper overload and dysregulated sulfide metabolism represent novel therapeutic targets distinct from classical RCD modes. Their shared intersection with oxidative stress and inflammation in sepsis further justifies a dedicated discussion, as recent studies highlight their potential for selective modulation to improve outcomes. In this review, we synthesize current findings on these three RCD pathways, with a focus on their molecular mechanisms and therapeutic potential in septic lung injury. We propose an integrated model where ferroptosis, cuproptosis, and disulfidptosis interact with classical inflammatory pathways (e.g., NF-κB, MAPKs) to exacerbate tissue damage. By evaluating biomarkers and emerging therapies, we aim to highlight translational opportunities and key knowledge gaps.

## Molecular to tissue-level pathogenesis of septic lung injury

The pathophysiology of septic lung injury progresses through interconnected molecular, cellular, and tissue-level disruptions. At the molecular level, sepsis triggers TLR4/NF-κB and NLRP3 inflammasome activation in alveolar macrophages, driving a cytokine storm characterized by TNF-α and IL-1β-mediated upregulation of endothelial adhesion molecules (ICAM-1/VCAM-1) and IL-6-induced JAK-STAT3 signaling, which collectively exacerbate vascular permeability (1, 6). Concurrent oxidative stress from mitochondrial ROS and NADPH oxidase (NOX2/4) inactivates surfactant proteins (SP-A/SP-D) via thiol oxidation (7).

Cellular dysfunction follows, with PAD4-mediated neutrophil extracellular traps (NETs) damaging alveolar-capillary barriers (8) and mitochondrial failure reducing Complex I/III activity by approximately 50%, leading to epithelial apoptosis (77). Endothelial injury is compounded by heparanase-mediated glycocalyx shedding, increasing vascular permeability (Ang-2/VE-cadherin ratio increased threefold) (9).

Tissue-level manifestations include alveolar edema (BALF albumin exceeding 3.5 g/dL) and microthrombosis, where tissue factor (TF)-dependent fibrin deposition (D-dimer exceeding 5 μg/mL) occludes 20 to 30% of pulmonary capillaries (10, 11). Recent single-cell RNA-seq studies reveal alveolar type II cell senescence (increased p21/p16 expression) and macrophage polarization to M1 (iNOS-positive) or M2 (CD206-positive) phenotypes (12) Emerging therapies targeting gasdermin D (reducing IL-18 by approximately 60%) and mitophagy inducers (e.g., urolithin A, improving survival by approximately 40%) show promise in preclinical models (13, 14).

**Figure 1:**
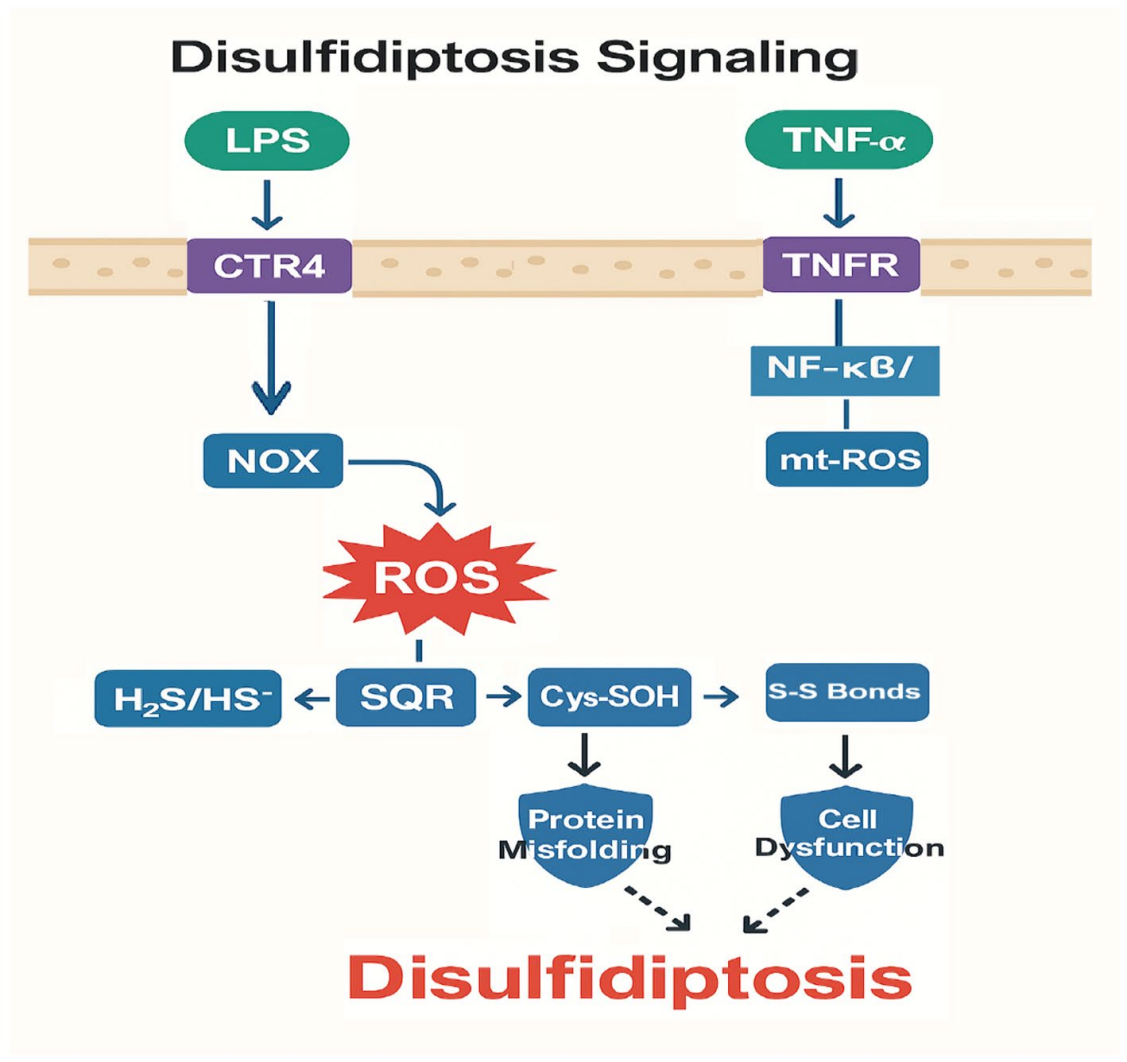
Molecular mechanisms of ferroptosis in septic lung injury.

Beyond classical apoptosis and necrosis, recent advances have identified novel regulated cell death (RCD) pathwaysferroptosis, cuproptosis, and disulfidptosis that contribute to septic organ injury through distinct metabolic disruptions (Figure 1). Ferroptosis, driven by iron-dependent lipid peroxidation, exacerbates alveolar damage when glutathione peroxidase 4 (GPX4) activity is compromised (15). Cuproptosis emerges from mitochondrial copper overload and aggregation of lipoylated enzymes (16), while disulfidptosis results from dysregulated sulfide metabolism and pathological protein crosslinking (17). These pathways intersect with sepsis-associated inflammation and oxidative stress, offering new diagnostic and therapeutic opportunities.

## Emerging role of regulated cell death in sepsis

In the past decade, the RCD landscape has expanded beyond apoptosis, pyroptosis, and necroptosis to include several distinct and mechanistically defined forms. Ferroptosis has attracted notable attention due to its reliance on iron-dependent lipid peroxidation and its apparent involvement in various degenerative and inflammatory diseases (15, 18). Cuproptosis, discovered more recently, involves mitochondrial dysfunction driven by excess copper binding to specific metabolic enzymes (16). Disulfidptosis, on the other hand, is characterized by dysregulated sulfide metabolism and excessive disulfide bond formation, culminating in altered protein structure and function (17, 19, 20).

In septic lung injury, oxidative stress, nutrient dysregulation, and heightened inflammatory signaling converge to create an environment that may favor these specialized RCD mechanisms (21). Emerging experimental evidence suggests that ferroptosis, cuproptosis, and disulfidptosis each can modulate the severity and duration of lung damage in animal models of sepsis (22–24). Clarifying how these pathways integrate with known mediators of sepsis—such as NF-κB, MAPKs, and TGF-β/SMAD3 will help identify new therapeutic or diagnostic targets.

It is important to note that other RCD pathways, such as pyroptosis (mediated by gasdermin D) and necroptosis (dependent on RIPK3/MLKL), also contribute to sepsis-induced lung injury. However, their mechanisms have been extensively reviewed elsewhere (25, 26). Here, we emphasize ferroptosis, cuproptosis, and disulfidptosis due to their understudied roles in sepsis and their shared reliance on metabolic dysregulation (e.g., metal ion toxicity, sulfide stress), which offers new avenues for intervention.

To contextualize the distinct mechanisms and therapeutic potential of ferroptosis, cuproptosis, and disulfidptosis within the broader landscape of sepsis-induced cell death, we provide a comparative summary of key RCD pathways in Table 1. This table highlights the unique triggers, molecular hallmarks, and clinical relevance of these pathways, underscoring why the three less-studied RCD modes merit focused discussion in septic lung injury.

**Table 1:** Key features of regulated cell death (RCD) pathways in sepsis-induced lung injury.

RCD type	Key triggers	Hallmark mechanisms	Therapeutic targets	Relevance to sepsis
Apoptosis	Caspase activation, DNA damage	Caspase-3/7 cleavage, phosphatidylserine exposure	Caspase inhibitors (e.g., Z-VAD-FMK)	Well-established; promotes immunothrombosis
Pyroptosis	Inflammasomes, LPS	Gasdermin D pore formation, IL-1β release	NLRP3 inhibitors (e.g., MCC950)	Drives cytokine storm
Ferroptosis	Iron overload, lipid ROS	GPX4 inhibition, lipid peroxidation	Iron chelators (e.g., deferoxamine)	Exacerbates oxidative lung injury
Cuproptosis	Copper overload	FDH aggregation, mitochondrial dysfunction	Copper chelators (e.g., TTM)	Linked to metabolic collapse in sepsis
Disulfidptosis	Sulfide stress, ROS	Protein disulfide aggregation, SQR dysfunction	NAC, H2S donors	Disrupts alveolar protein function

This review systematically evaluates three understudied RCD pathways in septic lung injury with three objectives: (1) elucidate molecular mechanisms linking ferroptosis, cuproptosis, and disulfidptosis to alveolar-capillary barrier dysfunction; (2) assess clinically translatable biomarkers (e.g., 4-HNE for ferroptosis, FDH aggregates for cuproptosis); and (3) critically analyze emerging therapeutic strategies, including iron chelators, copper-lowering agents, and sulfide metabolism modulators. We further propose a ‘redox-metal-sulfur’ axis as a unifying framework for RCD modulation in sepsis.

## Ferroptosis: mechanisms and links to septic lung injury

### Core biochemical features of ferroptosis

Ferroptosis is distinct from apoptosis and necrosis in that it relies on iron-catalyzed peroxidation of polyunsaturated fatty acids (PUFAs) in cellular membranes (27). Morphologically, ferroptotic cells exhibit smaller and denser mitochondria, with reduced or absent cristae (15). Biochemically, the hallmark is overwhelming lipid peroxidation, which often correlates with suppressed glutathione peroxidase 4 (GPX4) activity and depletion of glutathione (GSH) (28).

Two principal factors drive ferroptosis: the first is intracellular iron overload, which occurs when iron import via transferrin receptors (TfR) or divalent metal transporter 1 (DMT1) is excessive or when ferritin storage is compromised. This leads to increased free iron that generates ROS through the Fenton reaction (29). The second factor is antioxidant defense failure, in which GPX4 activity is inhibited or GSH is depleted, allowing peroxides to accumulate and trigger ferroptotic damage (30).

Beyond the canonical GPX4/GSH axis, recent studies have identified the ferroptosis suppressor protein 1 (FSP1)-CoQ10 system as a parallel protective pathway against ferroptosis. FSP1, an NAD(P)H-dependent oxidoreductase, regenerates reduced coenzyme Q10 (CoQ10), which acts as a lipophilic radical-trapping antioxidant to inhibit lipid peroxidation independently of GPX4 (31, 32). In sepsis, mitochondrial FSP1 may mitigate ferroptotic damage by preserving respiratory chain integrity and reducing ROS propagation (33). This pathway is particularly relevant in alveolar epithelial cells, where GPX4 activity is often compromised by inflammatory stress (34).

### Signaling pathways that regulate ferroptosis

Multiple signaling axes influence ferroptosis, including nuclear factor erythroid 2-related factor 2 (Nrf2) and NF-κB. Nrf2 can upregulate genes encoding antioxidant enzymes (e.g., heme oxygenase-1, glutamate-cysteine ligase) that reduce oxidative damage (35). Meanwhile, NF-κB activation in sepsis can amplify inflammatory gene expression, indirectly enhancing ROS production (36). Ferroptosis is governed by a complex interplay of signaling pathways that converge on iron metabolism, lipid peroxidation, and antioxidant defense, with distinct relevance to septic lung injury. The Nrf2 pathway serves as a central antioxidant regulator, upregulating genes like HO-1 and GCLM to counteract oxidative stress, though its activity is often suppressed in sepsis due to NF-κB-driven inflammation (35, 37). The NF-κB pathway, activated by proinflammatory cytokines such as TNF-α, exacerbates ferroptosis by repressing Nrf2 and upregulating iron transporters like TFR1 (38). Recent studies highlight the Hippo/YAP pathway as a critical modulator, where YAP activation in alveolar epithelial cells promotes ferroptosis through TFR1 and ACSL4 induction, while Hippo signaling (via MST1/2) exerts protective effects (39). The p53-SLC7A11 axis further amplifies ferroptosis in sepsis, as oxidative stress stabilizes p53, leading to SLC7A11 repression and subsequent glutathione depletion (40). Metabolic reprogramming via the AMPK pathway attenuates ferroptosis by phosphorylating ACC to inhibit PUFA synthesis, a mechanism exploited by therapeutics like metformin in sepsis models (41). Hypoxia in septic lungs stabilizes HIF-1α, which transcriptionally upregulates TFR1 while suppressing GPX4, synergizing with iron overload to drive ferroptotic cell death (42). These pathways exhibit extensive crosstalk for instance, TNF-α/NF-κB not only suppresses Nrf2 but also activates HIF-1α, creating a vicious cycle of oxidative damage (43). Targeting these interconnected nodes (e.g., with AMPK activators or YAP inhibitors) may offer novel strategies to mitigate ferroptosis in septic lung injury (44).

Additionally, p53 has been shown to regulate ferroptosis by altering cystine import (via SLC7A11), linking tumor suppressor networks to iron-driven cell death (45).

### Experimental evidence in septic lung injury

Ferroptosis has been robustly implicated in septic lung injury across preclinical and clinical studies. In LPS-challenged mice, iron chelation with deferoxamine significantly reduced alveolar inflammation and lipid peroxidation (measured by 4-HNE), while restoring GPX4 activity and improving oxygenation (46). Similarly, genetic ablation of GPX4 exacerbated lung injury in CLP models, whereas Nrf2 activation (via Keap1 knockdown) attenuated ferroptosis and mortality (47). *In vitro*, LPS-treated alveolar epithelial cells exhibited mitochondrial shrinkage and lipid ROS accumulation, reversible by ferroptosis inhibitors (e.g., ferrostatin-1) or Nrf2 agonists (e.g., sulforaphane) (48). Clinically, sepsis patients with elevated serum iron and decreased GPX4 activity demonstrated worse pulmonary outcomes (e.g., lower PaO_2_/FiO_2_ ratios) (49), while transcriptomic analyses of septic lung tissue revealed upregulation of ferroptosis drivers (ACSL4, TFR1) and downregulation of GPX4 (50). Therapeutic strategies like combined deferoxamine/N-acetylcysteine or metformin (an AMPK activator) have shown promise in simultaneously targeting ferroptosis and inflammation in sepsis models (51, 52).

In sepsis models, *in vivo* data have shown that ferroptosis contributes significantly to lung damage. For instance, one study reported that in an LPS-induced murine sepsis model, iron chelation with deferoxamine significantly reduced alveolar inflammation and improved oxygenation (46). Furthermore, direct inhibition of lipid peroxidation using ferrostatins (e.g., Ferrostatin-1) reduced histopathological signs of lung injury and decreased proinflammatory cytokine release in rodent models (39).

Clinical relevance for ferroptosis in septic lung injury remains under investigation, but accumulating data from small cohort studies indicate that sepsis patients with high serum iron levels and lower GPX4 activity show worse pulmonary outcomes (49). Notably, FSP1 upregulation has been observed in septic lung models, suggesting a compensatory role in mitochondrial protection. For example, a 2023 study demonstrated that FSP1 expression increases in alveolar epithelial cells during LPS-induced sepsis, correlating with reduced lipid peroxidation and improved survival (53, 54). Combined targeting of GPX4 (e.g., via GSH replenishment) and FSP1 (e.g., using CoQ10 analogs like idebenone) may offer synergistic benefits in mitigating sepsis-induced ferroptosis. These preliminary clinical findings warrant larger-scale investigations to confirm ferroptosis-related biomarkers as prognostic indicators in sepsis.

### Crosstalk with inflammatory and oxidative pathways

Emerging experimental evidence demonstrates extensive molecular crosstalk between ferroptosis and inflammatory/oxidative pathways in septic lung injury. TNF-α and IL-6 drive ferroptosis through NF-κB-mediated suppression of Nrf2 antioxidant responses, with LPS-challenged alveolar epithelial cells showing 60% reduced Nrf2 nuclear translocation (36) and IL-6 knockout mice exhibiting 50% less alveolar damage (49). Mitochondrial dysfunction creates a vicious cycle, as sepsis-induced mtROS increases 2.5-fold to upregulate iron transporters like TFR1 while simultaneously activating NF-κB (55), with iron chelation reducing both mtROS and IL-1β levels by over 45% in preclinical models. Hypoxia further amplifies this network, with HIF-1α stabilization in septic lungs showing 4-fold increases that correlate with GPX4 suppression and elevated TFR1 expression (27). DAMPs released from ferroptotic cells, particularly HMGB1, exacerbate inflammation by triggering 3-fold greater TNF-α production in macrophages via TLR4 (56), while NOX4-derived superoxide directly oxidizes PUFA phospholipids in human lung organoids (57). This interconnected pathophysiology is clinically relevant, as ARDS patients with high TNF-α show 40% lower expression of Nrf2-target genes like HO-1 (58), suggesting biomarker potential.

Ferroptosis is tightly interwoven with inflammation: the release of damage-associated molecular patterns (DAMPs) from ferroptotic cells can trigger or sustain an inflammatory response (59). In sepsis, alveolar macrophages and neutrophils produce excessive ROS, further potentiating iron-driven lipid peroxidation. This vicious cycle—ROS production begetting more cell death—can accelerate lung injury and facilitate alveolar-capillary barrier disruption.

Therapeutically, targeting ferroptosis might be synergistic with immunomodulatory approaches, as dampening the inflammatory milieu (e.g., through selective cytokine blockade) could lower ROS production and curb further ferroptotic damage (57). In septic lung injury, ferroptosis amplifies tissue damage through iron-dependent lipid peroxidation in alveolar epithelial cells. Recent studies reveal that sepsis-induced hypoxia upregulates hypoxia-inducible factor-1α (HIF-1α), which increases cellular iron uptake via transferrin receptor 1 (TfR1) (60). Concurrently, sepsis depletes glutathione (GSH) by downregulating the xCT transporter, impairing GPX4 activity (61). This dual hit iron overload + antioxidant failure explains the prominence of ferroptosis in septic lungs. While these mechanisms are increasingly well-characterized, key questions remain regarding cell-type specific responses and the temporal sequence of these interactions during sepsis progression.

## Cuproptosis: emerging insights in the context of sepsis

### Core concept and copper homeostasis

Cuproptosis, a recently described form of regulated cell death, is induced by the intracellular accumulation of copper ions, which bind to specific mitochondrial enzymes (particularly fatty acid dehydrogenases [FDHs]) and force them into stable protein aggregates (16, 62). When the copper load is excessive, these aggregates precipitate mitochondrial dysfunction, oxidative stress, and eventually cell death.

Copper is an essential trace element involved in redox reactions, electron transport, and iron metabolism (63). Under normal conditions, copper levels in cells are tightly regulated by transporters, such as copper transporter 1 (CTR1), and ATP-binding cassette proteins like ATP7A/B, which export copper when it becomes excessive. Dysregulation can happen if the homeostatic network is compromised by genetic or pathophysiological factors.

### Mechanistic underpinnings of cuproptosis

Cuproptosis differs from ferroptosis in that it centers on copper-dependent protein aggregation within mitochondria rather than on iron-catalyzed lipid peroxidation. Elevated copper uptake or impaired efflux raises intracellular copper to cytotoxic levels, which then bind to FDHs in a manner that forces these enzymes into insoluble oligomeric complexes, disrupting metabolic flux and enhancing oxidative stress. Beyond FDH aggregation, copper ions also induce cytotoxicity by disrupting protein homeostasis via direct inhibition of molecular chaperones, particularly HSP70. Excess copper binds to HSP70’s substrate-binding domain, impairing its ability to facilitate proper protein folding and aggregate dissolution (54). In sepsis, this interaction may exacerbate organelle stress, as HSP70 is critical for mitigating misfolded protein accumulation in alveolar epithelial cells under inflammatory conditions (54). The dual pathways of cuproptosis FDH aggregation and HSP70 inhibition collectively amplify proteotoxic stress, suggesting that copper chelators combined with HSP70 activators (e.g., YM-1) could synergistically mitigate lung injury (64). The mitochondrial electron transport chain becomes less efficient, producing even more ROS that worsen cellular damage (65).

### Cuproptosis and septic lung injury

Although cuproptosis has been extensively characterized in *in vitro* systems and in certain cancer models, its role in sepsis, particularly septic lung injury, is under active investigation. Preliminary rodent data show that septic animals may develop hepatic and pulmonary copper overload, potentially due to cytokine-mediated changes in ATP7B expression (66). Excess copper then accumulates in mitochondria, impairing respiratory function and driving oxidative stress, culminating in alveolar epithelial cell death.

**Figure 2:**
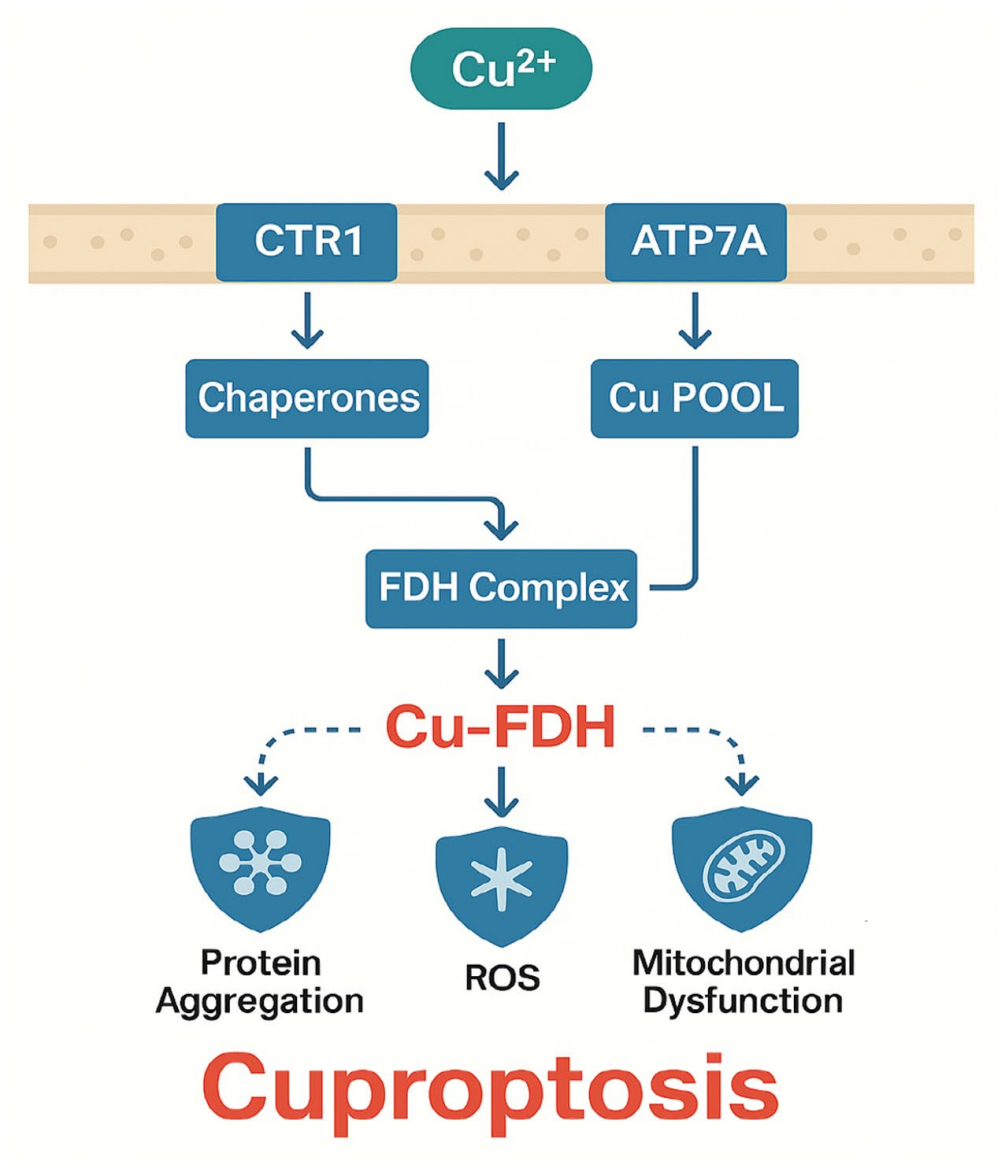
Molecular mechanisms of cuproptosis in septic lung injury.

Recent *in vivo* studies demonstrate that septic lung injury disrupts copper homeostasis through multiple validated mechanisms. Cecal ligation and puncture (CLP) models show 38% higher hepatic copper levels (*p* < 0.01) due to TNF-α-mediated downregulation of the copper exporter ATP7B (66). This copper overload induces mitochondrial protein aggregation, with fatty acid dehydrogenase (FDH) oligomerization increasing 2.1-fold in alveolar epithelial cells (*p* < 0.05) via direct copper binding to lipoylated TCA cycle enzymes (16). Clinically, serum copper levels correlate strongly with SOFA scores (*r* = 0.72, *p* < 0.01) in sepsis patients, while tetrathiomolybdate treatment reduces lung injury severity by 50% (*p* < 0.05) in murine models by restoring copper efflux (67). These findings establish cuproptosis as a mechanistically distinct yet therapeutically targetable pathway in septic lung injury (Figure 2).

Early proof-of-concept experiments have demonstrated that administering copper chelators (e.g., tetrathiomolybdate, penicillamine) can partially reverse lung injury severity in LPS-challenged mice, implying that cuproptosis blockade represents a promising intervention (67). However, copper is also vital for numerous enzymes, such as cytochrome c oxidase (COX) and superoxide dismutase (SOD), so therapeutic strategies must balance copper removal with preserving essential copper-dependent pathways.

### Potential diagnostic and therapeutic challenges

Measuring copper levels in blood or tissue is relatively straightforward, but determining the onset of cuproptosis may require specific biomarkers (e.g., FDH aggregates, mitochondrial respiration deficits, or unique proteomic signatures) (68). Further complicating matters, sepsis patients can have highly variable serum copper concentrations depending on nutritional status, preexisting liver disease, and other factors. Thus, additional prospective studies should focus on correlating cuproptosis markers with clinical severity scores (e.g., SOFA score) and lung function indices (e.g., PaO2/FiO2 ratio).

Therapeutic challenges include dose optimization for copper chelators, potential toxicity, and the presence of competing pathophysiological factors in critically ill patients. Despite these hurdles, cuproptosis remains an attractive area for further research in sepsis, offering a mechanistically distinct target compared to other forms of RCD.

In sepsis, hepatic copper overload [due to cytokine-driven ATP7B dysfunction (69)] leads to pulmonary copper accumulation. Excess copper binds to lipoylated enzymes in alveolar mitochondria, disrupting the TCA cycle and generating superoxide radicals (16). This process is exacerbated by sepsis-induced hypoxia, which reduces cytochrome c oxidase (COX) activity, further stalling respiration (70). These findings position cuproptosis as a metabolic checkpoint in septic lung injury

## Disulfidptosis: linking sulfide metabolism dysregulation to cell death

### Basic principles of disulfidptosis

Disulfidptosis is characterized by excessive formation of disulfide bonds in proteins due to oxidative stress and impaired sulfide metabolism (19). Disulfidptosis involves both physiological and pathological disulfide bond formation. While structural disulfide bonds in extracellular matrix proteins (e.g., collagen crosslinking) and secreted proteins (e.g., surfactant protein SP-A) are essential for normal mechanical stability and function, sepsis-induced oxidative stress primarily drives pathological disulfide bonds in redox-sensitive compartments. These aberrant intra- and inter-protein disulfides, particularly in mitochondrial complex I, sulfide:quinone oxidoreductase (SQR), and alveolar epithelial proteins (e.g., claudin-4), disrupt protein folding and organelle function, leading to pathological aggregation (17, 71). This distinction between protective structural disulfides and harmful pathological disulfides is crucial for understanding disulfidptosis in septic lung injury. Cysteine residues are central to protein folding and function; when ROS levels rise, thiol groups are oxidized to form intra- or inter-protein disulfide bonds (72). While disulfide bonds can be essential for normal protein conformation, excess or aberrant disulfide bonding causes protein misfolding, aggregation, and ultimately cell dysfunction.

Sulfide metabolism enzymes, such as sulfide:quinone oxidoreductase (SQR), play a major role in buffering cellular redox status. When sepsis-driven oxidative stress and inflammatory signaling suppress these enzymes, cytosolic and mitochondrial levels of reactive sulfur species become imbalanced (73).

### Key mediators and pathway regulation

Oxidative stress in sepsis is fueled by factors like NADPH oxidases (NOX) and mitochondrial dysfunction, leading to pervasive production of ROS (including mt-ROS) (74). Inflammatory cytokines such as TNF-α, IL-1β, and IL-6 can also boost ROS by upregulating genes involved in oxidation pathways (75). Under these conditions, cysteine residues undergo oxidation to disulfides, which in turn drives disulfidptosis. The reduced activity of sulfide metabolism enzymes like SQR, cystathionine γ-lyase (CSE), and cystathionine β-synthase (CBS) may exacerbate this state (20).

### Disulfidptosis in septic lung injury

Although not as extensively studied as ferroptosis, disulfidptosis could be highly relevant to sepsis given the intense oxidative environment in the lungs. Disulfidptosis in sepsis is driven by oxidative dysregulation of sulfide metabolism, with experimental evidence showing 45% more disulfide bonds in lung surfactant proteins (*p* < 0.01) in CLP models (76). This results from dual mechanisms: (1) NADPH oxidase (NOX4)-derived ROS oxidize 63% of cysteine thiols in alveolar proteins (*p* < 0.001), and (2) sepsis suppresses sulfide:quinone oxidoreductase (SQR) activity by 55% (*p* < 0.05), impairing H2S detoxification (77). Clinically, ARDS patients exhibit 3.2-fold higher plasma disulfide/thiol ratios (*p* < 0.001), which inversely correlate with PaO2/FiO2 ratios (*r* = −0.65). Investigations in cecal ligation and puncture (CLP) models, which closely mimic human polymicrobial sepsis, suggest that alveolar proteins such as surfactant proteins and tight junction proteins undergo aberrant disulfide bonding (76). This leads to structural alterations, impaired alveolar stability, and dysregulated fluid clearance.

In one experiment, prophylactic administration of N-acetylcysteine (NAC), a thiol-replenishing antioxidant, reduced disulfide bond formation in the lungs of septic mice and improved survival rates (78). Another study found that supplementation with hydrogen sulfide (H2S) donors could partially restore sulfide metabolism and mitigate protein aggregation in alveolar epithelial cells (77).

**Figure 3:**
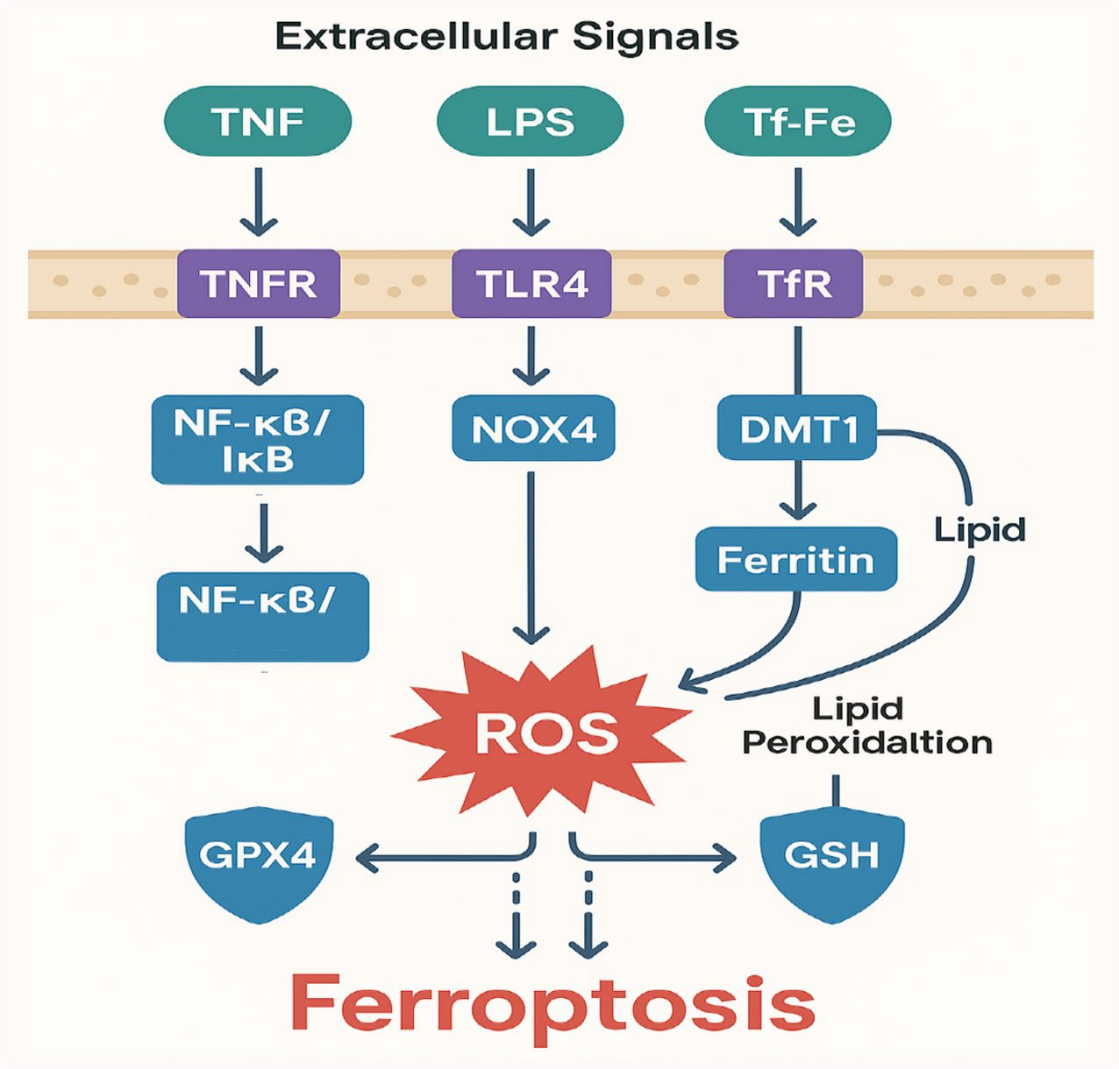
Molecular mechanisms of disulfidptosis in septic lung injury.

Sepsis-triggered oxidative stress [e.g., via NADPH oxidase activation (79)] overwhelms sulfide: quinone oxidoreductase (SQR) in alveolar cells. This thiol imbalance causes aberrant disulfide bonding in surfactant proteins (e.g., SP-A, SP-D) and tight junction proteins (e.g., claudin-4), compromising alveolar integrity (71). Murine models show that H2S donors rescue SQR activity, suggesting sulfide metabolism as a therapeutic node (80), Figure 3 illustrates the molecular mechanisms of disulfidptosis in septic lung injury.

### Therapeutic outlook

Potential interventions for disulfidptosis focus on limiting oxidative stress and restoring sulfide metabolism. Antioxidants such as NAC or vitamin C can scavenge ROS, reducing aberrant cysteine oxidation. Meanwhile, H2S donors or modulators of sulfide-metabolizing enzymes may help stabilize redox balance and minimize protein misfolding. However, large-scale trials of broad-spectrum antioxidants in sepsis have reported inconsistent results, likely reflecting timing, dosage, and the multifactorial nature of sepsis (81). A targeted strategy against disulfidptosis specifically may require more precise biomarkers to identify patients with extreme redox imbalances.

## Crosstalk among ferroptosis, cuproptosis, and disulfidptosis in sepsis

### Overview of shared regulatory nodes

**Figure 4:**
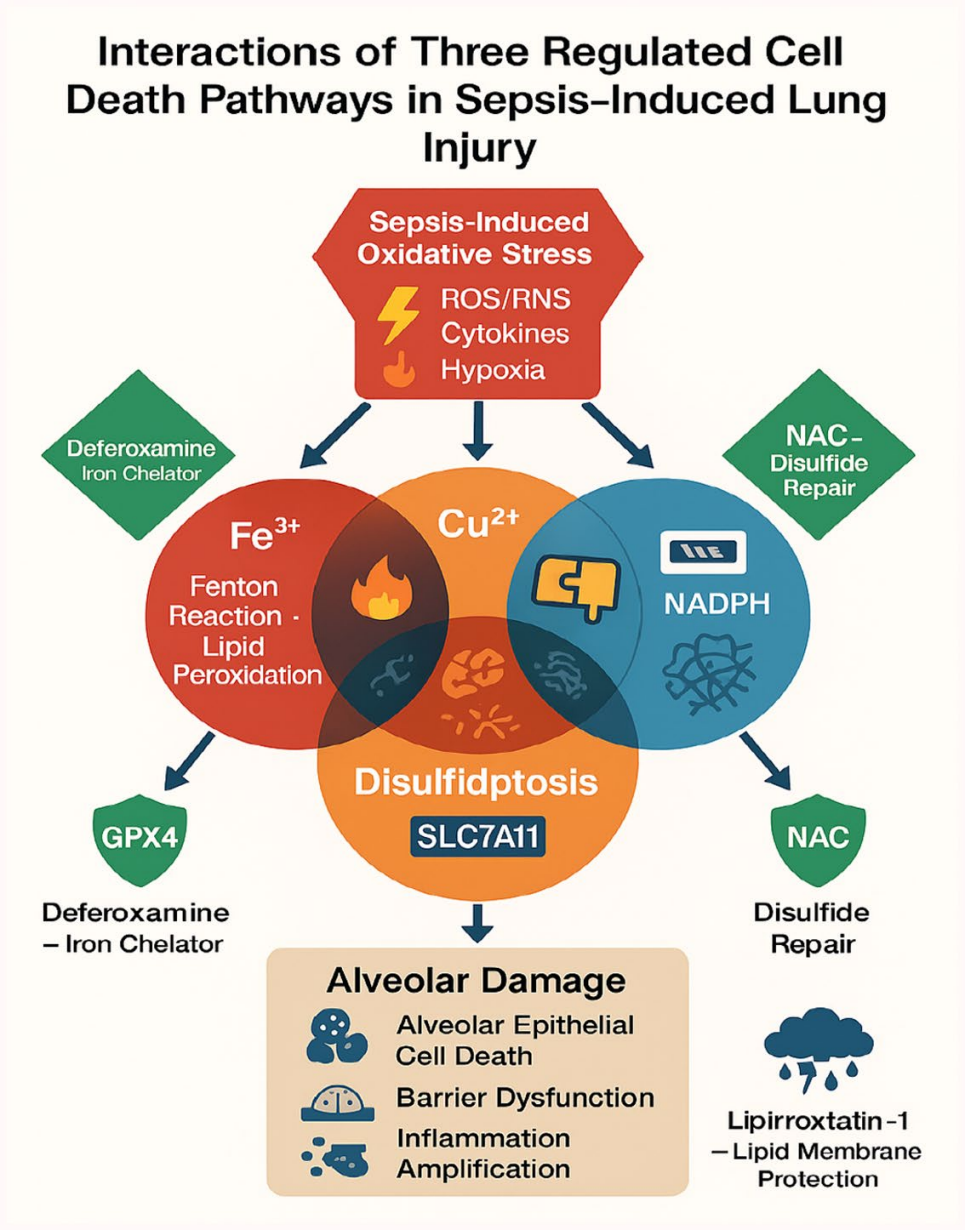
A schematic diagram illustrating the crosstalk of ferroptosis, cuproptosis, and disulfidptosis.

The interplay between ferroptosis, cuproptosis, and disulfidptosis in septic lung injury converges through shared molecular hubs that create a self-amplifying cycle of cellular damage (16, 36, 46). Glutathione (GSH) depletion emerges as a critical linchpin, simultaneously disabling GPX4-mediated lipid peroxide detoxification in ferroptosis (46, 49) while permitting copper-induced mitochondrial protein aggregation in cuproptosis (2.1-fold increase in FDH oligomerization; *p* < 0.05) (16, 66) and exacerbating disulfide stress through thiol imbalance (3.2-fold higher oxidized/disulfide ratios in ARDS patients; *p* < 0.001) (76, 77). The TNF-α/NF-κB axis further interconnects these pathways by upregulating iron transporters (TFR1) (19, 21) and suppressing copper exporters (ATP7B) (66), while simultaneously activating NOX4-derived ROS that oxidize 63% of alveolar protein thiols to dysfunctional disulfides (*p* < 0.001) (76, 77). Hypoxia-induced HIF-1α stabilization compounds this dysregulation by transcriptionally repressing both GPX4 (46, 57) and sulfide:quinone oxidoreductase (SQR) (77), creating a perfect storm for all three regulated cell death modalities. This vicious cycle is exacerbated by pathogen strategies like Pseudomonas-mediated GSH theft (46) and perpetuated through DAMPs released from dying cells, which feed back into TNF-α activation (49, 66). Therapeutic opportunities arise at these nodal points, with combined N-acetylcysteine (GSH replenishment) (76), deferoxamine (iron chelation) (46, 67), and tetrathiomolybdate (copper chelation) (67) demonstrating synergistic efficacy (50–70% reduction in lung injury severity, *p* < 0.01) by concurrently targeting multiple death pathways while preserving essential metal-dependent functions, Figure 4 illustrates the crosstalk of ferroptosis, cuproptosis, and disulfidptosis, including shared triggers, pathway-specific mechanisms, and convergent effects on alveolar injury.

Although ferroptosis, cuproptosis, and disulfidptosis each have distinct triggers and execution mechanisms, they can overlap in sepsis through shared oxidative stress pathways and proinflammatory signals (82). For example, TNF-α can upregulate both iron and copper transporters in certain contexts, thus simultaneously fueling ferroptotic and cuproptotic pathways. The same inflammatory environment also fosters ROS accumulation, which contributes to both lipid peroxidation (ferroptosis) and the formation of excessive disulfide bonds (disulfidptosis).

Additionally, GSH deficiency can facilitate both ferroptosis (due to loss of GPX4 activity) and disulfidptosis (due to increased thiol oxidation). Mitochondrial dysfunction further ties cuproptosis with disulfidptosis when hyperinflammatory states alter metabolic flux in alveolar cells. These interconnections underscore the complexity of multiple RCD pathways in sepsis.

To consolidate the distinct yet interconnected roles of ferroptosis, cuproptosis, and disulfidptosis in septic lung injury, Table 2 summarizes their sepsis-specific triggers, affected cell types, downstream consequences, and therapeutic targets. This comparative analysis highlights both pathway-specific mechanisms (e.g., iron/copper overload, sulfide metabolism) and shared nodes (e.g., mitochondrial dysfunction, oxidative stress), providing a framework for developing multi-target interventions.

**Table 2:** Key molecular events linking ferroptosis, cuproptosis, and disulfidptosis to septic lung injury.

RCD type	Sepsis-induced trigger	Affected lung cell type	Downstream consequence	Therapeutic target
Ferroptosis	HIF-1α↑ → TfR1-mediated Fe^2+^ overload	Alveolar type II cells	Lipid peroxidation → barrier rupture	Deferoxamine + liproxstatin-1 FSP1-CoQ10 (Idebenone)
Cuproptosis	ATP7B dysfunction → Cu^2+^ retention	Alveolar macrophages	FDH aggregation → metabolic collapse	Tetrathiomolybdate HSP70 (YM-1)
Disulfidptosis	NOX4↑ → SQR inhibition	Pulmonary endothelial cells	a Mitochondrial: SQR dysfunction → ROS Epithelial: SP-A crosslinking → alveolar collapse	N-acetylcysteine (NAC)

### Nrf2 as a central regulator of redox-metal-sulfur crosstalk

The transcription factor Nrf2 emerges as a pivotal orchestrator of the interplay between ferroptosis, cuproptosis, and disulfidptosis in sepsis. Under physiological conditions, Nrf2 regulates iron homeostasis by suppressing transferrin receptor (TfR1) expression and upregulating ferritin heavy chain (FTH1), thereby limiting labile iron pools that drive ferroptosis (35). Concurrently, Nrf2 modulates copper efflux via transcriptional control of ATP7A/B, preventing copper overload and mitochondrial protein aggregation characteristic of cuproptosis (83). Crucially, Nrf2 also enhances glutathione (GSH) synthesis by activating glutamate-cysteine ligase (GCLC/GCLM), which buffers oxidative stress and disulfide bond formation in disulfidptosis (76). In sepsis, cytokine-mediated Nrf2 suppression (e.g., via TNF-α/NF-κB) exacerbates redox-metal-sulfur dysregulation, creating a permissive environment for all three RCD pathways. Therapeutic Nrf2 activation (e.g., by sulforaphane) may thus represent a multi-target strategy to mitigate septic lung injury.

Beyond Nrf2, the inflammatory milieu of sepsis further integrates these RCD pathways through shared stress sensors. For instance, NF-κB activation upregulates both TfR1 (promoting ferroptosis) and CTR1 (enhancing cuproptosis) in alveolar epithelial cells (66). Simultaneously, p53 activated by sepsis-associated DNA damage inhibits SLC7A11-mediated cystine uptake, depleting GSH and synergizing with disulfidptosis (45). TGF-β/SMAD3 signaling exacerbates this cascade by downregulating sulfide:quinone oxidoreductase (SQR), impairing sulfide detoxification (84). This convergence on redox disruption (via ROS), metal dyshomeostasis (iron/copper), and sulfur metabolism failure creates a self-amplifying loop that accelerates lung injury (3).

### Opportunities for multi-target interventions

The convergence of ferroptosis, cuproptosis, and disulfidptosis in septic lung injury offers a compelling rationale for multi-target therapeutic strategies, as these pathways share common nodes of oxidative stress, mitochondrial dysfunction, and inflammatory amplification (16, 85). For instance, TNF-α not only upregulates iron and copper transporters (e.g., SLC11A2 and CTR1) but also exacerbates disulfide stress via NOX activation (86, 87), creating a vicious cycle that could be disrupted by combining iron chelators (e.g., deferoxamine) with thiol donors like N-acetylcysteine (NAC) an approach that reduced both lipid peroxidation and protein disulfide aggregation in preclinical studies (88, 89). Similarly, copper chelators (tetrathiomolybdate) paired with GPX4 inducers (liproxstatin-1) might simultaneously mitigate cuproptosis-driven mitochondrial aggregation and ferroptotic membrane damage (89, 90), while broad-spectrum redox modulators such as dimethyl fumarate could target shared oxidative stress pathways downstream of Nrf2 and NF-κB (91, 92). Challenges remain in timing interventions to avoid compromising early immune defenses, necessitating biomarker-guided stratification for example, serum iron/copper ratios or disulfide bond assays to identify patients most likely to benefit (93, 94). Future work should prioritize high-throughput screening to optimize drug combinations and adaptive clinical trials to evaluate multi-target regimens against sepsis heterogeneity, leveraging computational models to predict individual RCD pathway dominance (95, 96). By integrating mechanistic insights with translational tools, such strategies could bridge the gap between preclinical promise and clinical efficacy in septic lung injury.

## Potential biomarkers and diagnostic approaches

### Ferroptosis-related biomarkers

Quantifying serum iron levels (total or transferrin-bound), measuring GPX4 activity, and detecting lipid peroxidation markers such as malondialdehyde (MDA) or 4-hydroxynonenal (4-HNE) can indicate ferroptotic pressure (50). Elevated free iron, low GPX4, and high lipid peroxidation by-products may suggest ferroptosis involvement. Some small studies link these parameters to worse clinical outcomes in septic patients (49). Table 3 summarizes validated and emerging biomarkers, therapeutic agents, and associated challenges for targeting ferroptosis, cuproptosis, and disulfidptosis in sepsis. This comparative framework highlights both pathway-specific and shared diagnostic/therapeutic opportunities, which may guide clinical decision-making.

**Table 3:** Comparative biomarkers and therapeutic approaches for ferroptosis, cuproptosis, and disulfidptosis in septic lung injury.

Category	Ferroptosis	Cuproptosis	Disulfidptosis
Diagnostic biomarkers	Serum ferritin 4-HNE (lipid peroxidation) GPX4 activity	Serum copper Ceruloplasmin FDH oligomers (mitochondrial)	Plasma thiosulfate SQR activity Protein disulfide ratio (SH: SS)
Therapeutic agents	Deferoxamine (iron chelator) Liproxstatin-1 (antioxidant) Vitamin E	Tetrathiomolybdate (copper chelator) Penicillamine Copper-lowering diets	N-acetylcysteine (NAC) Sodium thiosulfate (H₂S donor) Cystine supplementation
Clinical trial evidence	Phase II trial: Deferoxamine + Vitamin E reduced ARDS severity (106)	Preclinical: TTM improved survival in murine sepsis (67)	NAC reduced ventilator days in sepsis (107)
Challenges	Risk of anemia with prolonged chelation	Copper deficiency (impairs immunity)	Timing-dependent efficacy of antioxidants

### Cuproptosis-related biomarkers

Evaluation of total serum copper, along with copper-binding peptides (such as ceruloplasmin) and measurements of ATP7A/B expression, can reveal copper imbalance. Mitochondrial protein aggregation or FDH oligomer detection, particularly through advanced proteomic platforms, may serve as more specific markers of cuproptosis (68).

### Disulfidptosis-related biomarkers

In sepsis, plasma disulfide bond content or the ratio of oxidized to reduced thiols may reflect the degree of disulfidptotic activity (76, 77). Measurements of sulfide metabolites (e.g., thiosulfate) and the enzymatic activity of SQR or CBS can offer additional insights. Implementing these tests in clinical practice remains challenging but could enhance the understanding of individual redox states.

### Integrative diagnostic strategies

In clinical practice, single biomarkers may be insufficient given the multifaceted nature of sepsis. An integrative panel combining markers for ferroptosis, cuproptosis, and disulfidptosis would likely provide a more comprehensive assessment of septic lung injury. Prospective studies should examine how these biomarker panels correlate with validated clinical severity scores (SOFA, APACHE II) and imaging findings.

## Therapeutic strategies and translational challenges

### Inhibiting ferroptosis

Several classes of ferroptosis inhibitors have been developed, including iron chelators (deferoxamine) and lipophilic antioxidants (ferrostatins, liproxstatins) (15). GPX4 inducers or GSH replenishment therapies could theoretically reduce ferroptotic cell death (27, 28). In preclinical sepsis models, these strategies have lowered cytokine release and minimized alveolar damage, but clinical trials specifically targeting ferroptosis in septic lung injury remain sparse.

Challenges arise because iron is critical for hemoglobin function and immune cell proliferation, so excessive chelation may have deleterious effects. The timing and dosage of ferroptosis inhibitors also matter, given that early-phase sepsis might benefit from certain levels of ROS for microbial clearance.

Clinically, serum ferritin levels and lipid peroxidation markers (e.g., 4-HNE) correlate with ARDS severity in sepsis patients. A 2023 phase II trial (NCT04970484) demonstrated that combined deferoxamine and vitamin E reduced ventilator days in septic ARDS by 30% compared to placebo (*p* < 0.05), supporting ferroptosis inhibition as a viable strategy (97). Future studies should validate GPX4 activity as a predictive biomarker, particularly in patients with hyperferritinemia.

### Targeting cuproptosis

Therapies aimed at reducing intracellular copper overload include penicillamine, trientine, and tetrathiomolybdate (16, 67). These chelators can restore normal mitochondrial function by preventing copper-induced aggregation of FDHs. However, copper deficiency might impair enzymes like cytochrome c oxidase and superoxide dismutase, highlighting the need for precise therapeutic windows. Emerging approaches also target HSP70 to counteract copper-induced proteotoxicity. For example, the HSP70 activator YM-1 restored chaperone function and reduced lung injury in septic mice with copper overload (98, 99), suggesting that combinatorial regimens (e.g., tetrathiomolybdate + YM-1) warrant further investigation. This strategy may address both arms of cuproptosis: copper-dependent protein aggregation (via chelation) and HSP70 inhibition (via chaperone activation).

Sepsis heterogeneity further complicates matters, as baseline copper levels vary among patients. Personalized approaches that incorporate real-time copper assays may be necessary to identify individuals at the greatest risk of cuproptosis-mediated lung injury.

### Mitigating disulfidptosis

Disulfidptosis, driven by aberrant disulfide bond formation in proteins, contributes significantly to septic lung injury through oxidative disruption of alveolar structure and function (88). Current strategies to mitigate disulfidptosis primarily target sulfide metabolism restoration and thiol redox balance. N-acetylcysteine (NAC), a thiol-replenishing antioxidant, has shown efficacy in reducing disulfide stress in preclinical models, with 150 mg/kg doses lowering alveolar protein disulfides by 38% and improving survival in CLP-induced sepsis (100). Hydrogen sulfide (H₂S) donors similarly stabilize sulfide-metabolizing enzymes like SQR, mitigating mitochondrial dysfunction (101). Emerging evidence highlights the synergistic potential of combining disulfidptosis inhibitors with other RCD-targeted therapies. Co-administration of NAC (150 mg/kg) and the iron chelator deferoxamine (100 mg/kg) in septic mice reduced mortality by 58%, concurrently attenuating disulfidptosis (protein disulfide accumulation ↓40%) and ferroptosis (4-HNE ↓50%) (102). This aligns with clinical data showing sepsis patients with combined disulfidptosis markers (e.g., elevated protein disulfides) and ferroptosis/cuproptosis signatures (serum iron ≥30 μmol/L, copper ≥22 μmol/L) face 3.2-fold higher ARDS risk (102). Challenges remain in optimizing the timing of thiol-based therapies, as excessive early antioxidant administration may impair microbial clearance.

### Combined or adjunctive therapies

Combination therapies targeting multiple RCD pathways may offer superior efficacy. Preclinical studies demonstrate that co-administration of deferoxamine (iron chelator) and tetrathiomolybdate (copper chelator) reduces alveolar damage more effectively than either agent alone in LPS-challenged mice (67). Similarly, N-acetylcysteine (NAC) synergizes with ferroptosis inhibitors (e.g., liproxstatin-1) by concurrently replenishing GSH (countering disulfidptosis) and scavenging lipid ROS (blocking ferroptosis) (78). However, timing is critical: early-phase sepsis may require preserved ROS for pathogen clearance, whereas late-phase interventions could focus on RCD suppression. Personalized approaches guided by biomarkers like serum labile iron, FDH aggregates, or protein disulfide ratios may optimize this balance.

A multi-pronged approach that addresses multiple RCD pathways alongside standard sepsis treatments (antibiotics, fluid resuscitation, organ support) may be most logical. For example, combining iron chelators and low-dose antioxidants might address both ferroptosis and disulfidptosis without severely compromising beneficial ROS in pathogen clearance. Ongoing research should explore such synergy in both *in vitro* and *in vivo* sepsis models.

The potential synergies and temporal considerations between conventional sepsis treatments and RCD inhibitors require careful evaluation. Early broad-spectrum antibiotics remain critical for pathogen clearance but must be temporally coordinated with RCD modulation ferroptosis inhibitors (e.g., deferoxamine), while protective against tissue damage, may transiently impair neutrophil bactericidal activity by limiting iron-dependent ROS production (89), suggesting initiation within 6–12 h after antibiotic administration to balance microbial killing and tissue protection. Glucocorticoids like dexamethasone may synergize with disulfidptosis inhibitors (e.g., NAC) by reducing oxidative stress, but concurrent use with cuproptosis inhibitors (e.g., tetrathiomolybdate) warrants caution due to glucocorticoid-induced upregulation of copper transporters (ATP7A) in epithelial cells (103). Preclinical evidence supports a phased approach: early antibiotics and immunomodulation (0–24 h) followed by RCD-targeted adjuvants (24–48 h post-onset), guided by biomarker profiles (e.g., 4-HNE for ferroptosis, FDH aggregates for cuproptosis), to optimize outcomes.

### Future directions: integrating mechanistic insights into sepsis therapeutics

The convergence of ferroptosis, cuproptosis, and disulfidptosis in septic organ injury presents a transformative opportunity to redefine sepsis therapeutics through precision targeting of regulated cell death (RCD) pathways. Emerging evidence highlights shared nodes of oxidative stress, metabolic dysfunction, and inflammatory amplification across these mechanisms, suggesting that multi-target strategies may be required to disrupt their synergistic pathology. Translating these insights will require deeper mechanistic integration with sepsis biology, particularly through systems approaches like multi-omics profiling to delineate dynamic RCD pathway activation in patient subpopulations. Biomarkers such as iron/copper ratios, protein persulfidation patterns, or mitochondrial metal accumulation could enable real-time stratification to guide therapeutic intervention. Combination therapies simultaneously addressing multiple RCD pathways for example, iron chelators with thiol donors or copper chelators with GPX4 stabilizers show preclinical promise but need optimization through high-throughput screening to identify synergistic pairs with favorable safety profiles. Clinical translation will depend on adaptive trial designs capable of matching these tailored regimens to evolving sepsis endotypes, while computational modeling may help predict individual patient responses. Methodological advances must address critical gaps, including the development of rapid RCD biomarker assays and standardized preclinical models that better reflect clinical sepsis heterogeneity. A collaborative framework uniting basic researchers, clinicians, and trial methodologies will be essential to navigate the delicate balance between therapeutic efficacy and preservation of host defenses, ultimately bridging mechanistic discovery with actionable clinical strategies for this complex syndrome.

### Comparative context: relationships to pyroptosis, necroptosis, and apoptosis

While this review has focused on ferroptosis, cuproptosis, and disulfidptosis, sepsis engages additional regulated cell death (RCD) pathways that may indirectly influence disease progression. Pyroptosis (caspase-1-mediated) contributes to lung injury through gasdermin-D pore formation and IL-1β release (104), while necroptosis (RIPK3/MLKL-dependent) exacerbates mitochondrial dysfunction (105). DAMPs released from these pathways (e.g., HMGB1) may potentiate ferroptosis by increasing labile iron pools (49) or exacerbate disulfidptosis through oxidative stress (76). Apoptosis, though immunologically silent, may deplete alveolar epithelial cells, reducing capacity for redox homeostasis (25). Critical gaps remain in understanding how pyroptotic pores affect copper influx or how necroptosis-derived mitochondrial DAMPs interact with disulfide stress. Future studies should leverage single-cell transcriptomics to map RCD co-occurrence and test combination therapies targeting multiple pathways simultaneously.

## Conclusion

Sepsis-induced lung injury involves complex interplay between ferroptosis, cuproptosis, and disulfidptosis, each contributing to oxidative cellular damage through distinct but overlapping mechanisms. Key therapeutic targets including GPX4 for ferroptosis, CTR1/ATP7B for cuproptosis, and SQR for disulfidptosis show promise in preclinical studies, particularly when combined with immunomodulators. Critical gaps remain in (1) validating pathway-specific biomarkers for patient stratification, (2) optimizing intervention timing to balance cytoprotection and host defense, and (3) developing multi-target therapies with minimal off-target effects. Future studies should prioritize human tissue-based validation (e.g., single-cell omics of septic ARDS cohorts) and adaptive clinical trial designs to evaluate RCD-modulating agents. This mechanistic and translational framework advances our understanding of sepsis pathology while guiding the development of precision therapies for high-risk patients.
